# Restoring a critically endangered grassland orchid by co-planting to improve pollination and selecting sites based on pollinator availability

**DOI:** 10.3389/fpls.2025.1566543

**Published:** 2025-05-23

**Authors:** Noushka Reiter, Richard Dimon, Björn Bohman, Michael Batley, Alex McLachlan, John Woodward, Ryan D. Phillips

**Affiliations:** ^1^ Royal Botanic Gardens Victoria, Science Division, Cranbourne, VIC, Australia; ^2^ Department of Ecological, Plant and Animal Sciences, and Research Centre for Future Landscapes, La Trobe University, Melbourne, VIC, Australia; ^3^ Ecology and Evolution, Research School of Biology, The Australian National University, Canberra, ACT, Australia; ^4^ Research Centre for Ecosystem Resilience, Botanic Gardens of Sydney, Sydney, NSW, Australia; ^5^ Queensland Alliance of Agriculture and Food Innovation, University of Queensland, St Lucia, QLD, Australia; ^6^ School of Molecular Sciences, the University of Western Australia, Crawley, WA, Australia; ^7^ Department of Plant Protection Biology, the Swedish University of Agricultural Sciences, Lomma, Sweden; ^8^ Australian Museum Research Institute, Australian Museum, Sydney, NSW, Australia

**Keywords:** pollinator, bee, orchid, *Diuris*, restoration, reproduction, *APIs*, conservation

## Abstract

In many geographic regions grasslands have been heavily cleared and degraded, which represents a challenge for translocating threatened flora back into these landscapes. As most plant species require animals for pollination, pollinators are potentially a key limitation for re-establishing populations. For the Critically Endangered orchid *Diuris fragrantissima*, we identify the pollinator(s), survey for pollinators at candidate translocation sites, test if remnant size affects bee species richness, and test if pollination rates can be enhanced through co-planting with rewarding plant species. We found that *D. fragrantissima* is visited by ten species of bee but is only effectively pollinated by two native species, *Lipotriches (Austronomia)* sp. (Halictidae) and *Lasioglossum (Chilalictus) orbatum* (Halictidae), and the introduced honeybee *Apis mellifera* (Apidae). Interestingly, *A. mellifera* was responsible for the greatest number of pollinia removals and depositions. Pollinators of *D. fragrantissima* were not detected at some candidate translocation sites, with bee species richness and overall abundance significantly increasing with grassland remnant size. The pollination of *D. fragrantissima* was significantly enhanced through the presence of *Wahlenbergia stricta* (Campanulaceae) within 30 cm of plants, but not *Arthropodium strictum* (Asparagaceae) or *Dianella reflexa* (Asphodelaceae). We recommend that prior to conservation translocations of *Diuris* that pollinator surveys are undertaken, with preference given to larger grassland remnants. *Apis mellifera* may serve to buffer *D. fragrantissima* against loss of native pollinators from remnant grassland but could have adverse effects on other native species. We show that co-planting with rewarding species may be an effective approach for improving pollination success of threatened orchids.

## Introduction

In many geographic regions temperate grassland ecosystems have been adversely impacted by a combination of clearing for agriculture (Richter and Osborne, 2014; Mark and McLennan, 2005; Carbutt et al., 2017), degradation through altered management (Veldman et al., 2015; Sühs et al., 2020; Bardgett et al., 2021), and invasion by weed and shrubs (Eldridge et al., 2011; Van Auken, 2009; Archer et al., 2011). In temperate grasslands across six continents this has led to many species of endemic grassland flora becoming threatened (Linusson et al., 1998; Austrheim et al., 1999; Cousins and Eriksson, 2001; Silcock and Fensham, 2018). Reinstating threatened flora back into grasslands will require appropriate habitat management (Andrade et al., 2016; WallisDeVries et al., 2002) combined with conservation translocations. In conservation translocations, seed or propagated plants are used to bolster existing populations or found new insurance populations, as part of an overall aim of reducing extinction risk to a threatened species (IUCN/SSC, 2013). As most plant species require animals for pollination (Ollerton et al., 2011), a key element of habitat suitability for most plant species is the presence of suitable pollinator species (Phillips et al., 2020). Therefore, when translocating plants into grasslands, one needs to consider selecting sites where appropriate pollinators are present and managing the site to support pollinators. However, pollinators are typically not accounted for when selecting sites for conservation translocation (Silcock et al., 2019), which may contribute towards low rates of translocation success in some plant groups (e.g. Reiter et al., 2016).

Accounting for pollinators in conservation translocations may be most important for plants with relatively specialised pollination systems, as the patchy distribution of specific pollinators in the landscape means not all areas can support translocated plants. For example, when translocating an endangered orchid species, Reiter et al. (2017) had to survey over 100 sites to locate bushland that was of the correct vegetation association to support the threatened orchid, but where the pollinating wasp was also present. This issue is likely to be exacerbated in fragmented landscapes or areas of poorly managed habitat, as the focal pollinator species is likely to be lost from certain remnant patches of habitat (e.g. Pauw and Hawkins, 2011; Phillips et al., 2015). Given the evidence for reduced diversity and abundance of some pollinator groups in small habitat remnants (e.g. Steffan-Dewenter, 2003; Meneses Calvillo et al., 2010; Williams, 2011) and degraded grassland areas (Kwaiser and Hendrix, 2008; Sexton and Emery, 2020), plants with multiple pollinator species may also experience low pollinator availability through declining pollinator populations.

When pollinators are present at a candidate translocation site, there is the potential to manage the site to increase the reproductive success of the focal plant species. For threatened herbs, which often have a smaller display or lower nectar/pollen reward relative to other growth forms, one possible approach is to co-plant with food plants for the pollinator species. Having rewarding plants nearby can lure pollinators into an area, which then service co-occurring rewardless plants (Thomson, 1982; Laverty, 1992; Johnson et al., 2003; Peter and Johnson, 2008). However, there are also examples where co-occurring rewarding plants outcompete the focal plant in the attraction of pollinators, leading to reduced reproductive success (Lammi and Kuitunen, 1995; Internicola et al., 2006). As such, for threatened species conservation programs, there is a need to experimentally evaluate the consequences of co-planting before implementing this approach. Co-planting with rewarding plants may be a particularly effective strategy for grasslands, where the rewarding plants are predominantly herbaceous and can reach flowering age in a relatively short period of time in cultivation.

Southern Australia has a diverse terrestrial orchid flora, characterised by a high incidence of rarity (Phillips et al., 2011; Jones, 2021) and numerous specialised pollination systems (see data reviewed in Ackerman et al., 2023). In particular, the grasslands of south-eastern Australia contain a large proportion of threatened species, as most of the grasslands have been converted to pasture or cleared entirely, with between 76 and 100% decline in the extent of natural temperate grassland communities since European colonisation (Morgan and Williams, 2015; Lunt, 1994). However, due to weak implementation of conservation legislation and polices (Victorian Auditor-General’s Office; 2020), most remaining grasslands in this region are further threatened by weed invasion, altered fire regimes, climate change and extensive grazing by introduced herbivores (Morgan and Williams, 2015; Driscoll et al., 2019). One of the most threatened grassland orchid genera is *Diuris*, where members of the *D. chryseopsis* and *D. punctata* complexes are primarily grassland dwelling (Jones, 2021). *Diuris* contains 29 threatened species listed under state and/or national legislation. Among *Diuris*, there is evidence of specialised pollination systems in subgenus *Xanthodiuris* sect. *Xanthodiuris* and *Hesperodiuris* where pollinating *Trichocolletes* (Colletidae) bees are attracted through a general resemblance to a guild of co-occurring Fabaceae (Beardsell et al., 1986; Indsto et al., 2006; Scaccabarozzi et al., 2018, 2020). Alternatively, in subgenus *Xanthodiuris* sect. *Pendunculatae*, pollination of *D. chryseopsis* occurs by a range of bee species, particularly in the genus *Lasioglossum* (Halictidae), but without the *Diuris* bearing any close morphological resemblance to co-occurring food plants (Grinter, 2023). At present, there are relatively few detailed pollination studies of *Diuris*, but the precedent for both specialised and generalised pollination systems make it difficult to predict the importance of pollinator availability for selection of translocation sites. Of the four species tested for nectar to date, three species are nectarless (*Diuris maculata*, subgenus *Xanthodiuris* Indsto et al., 2006; *Diuris aurea*, subgenus *Hesperodiuris* Indsto et al., 2007; *Diuris magnifica*, subgenus *Hesperodiuris* Scaccabarozzi et al., 2020; for an exception see *Diuris alba*, Indsto et al., 2007; subgenus *Diuris*), meaning that co-planting with rewarding plants is a potential strategy to increase reproductive success. Indeed, in *Diuris brumalis*, there is evidence for increased reproductive success in the presence of the pollinating bee’s food plants, though in this instance the trend could arise through either magnet effects or more effective deception of the pollinators when the rewarding model flowers were relatively more abundant than the mimetic orchid (Scaccabarozzi et al., 2018).

*Diuris fragrantissima* (subgenus *Diuris*) is a Critically Endangered orchid that is endemic to the grasslands of the eastern Victorian Volcanic Plains (VVP), a region that contains over 60 species of flora listed as threatened under the Victorian Flora and Fauna Guarantee Act (1988). The remaining wild site is within the suburbs of the city of Melbourne. While no data are available on the pollinators of *D. fragrantissima*, *Exoneura* bees (Apidae) remove pollinia from the related species *D. alba* (Indsto et al., 2007), suggesting that *D. fragrantissima* is probably bee pollinated. *Diuris fragrantissima* was formerly locally common on the grasslands of the VVP to the west of Melbourne (AVH, 2024). As recently as 1966 *D. fragrantissima* was known from six sites (Jones, 2021). However, due to extensive degradation and destruction of the grasslands of the VVP (Morgan and Williams, 2015), *D. fragrantissima* now occurs only at one natural site. This site contains approximately 30 naturally occurring individuals (Duncan and Moloney, 2018), plus approximately 330 propagated plants (Karen Lester pers. comm.). In addition, there are two populations established via conservation translocation, but persistence at these sites is uncertain given a long history of failed translocations in this species (Duncan and Moloney, 2018). In the past, rates of fruit set were consistently low at the translocation site (5%, 2006-2013; Duncan and Moloney, 2018), and more recently (2018–2020) at both the wild supplemented site and translocation site (4.85 ± 3.03 SE % calculated from data in Duncan, 2021). Our study had two objectives; to more effectively select conservation translocation sites for *D. fragrantissima* and investigate options for improving reproductive success. Specifically, for *D. fragrantissima* we aim to: (1) identify the pollinator(s); (2) determine the presence of the pollinators at the wild sites, existing translocations and potential translocation sites; (3) investigate if low numbers of pollinators or diversity of potential pollinators are related to size of the remnant grassland and (4) investigate if pollination can be enhanced through the presence of co-flowering plants.

## Methods

### Study species

While the original decline of *D. fragrantissima* likely occurred due to the conversion of native grasslands into pasture, urban sprawl on the western side of Melbourne means that this area is now comprised of housing, industry and small areas of degraded grassland. Given its small geographic range, attractive flowers, and close proximity to Melbourne, there is a long history of attempted conservation actions with *D. fragrantissima*. Introductions have been undertaken with this species in 1950, 1982-1985, 2004 to 2005 (Murphy et al., 2008) and 2019-2023 (of an additional > 800 plants Karen Lester pers. comm.) in a 50-ha area of degraded remnant grasslands (site LA). All translocations up until 2005 failed (Duncan and Moloney, 2018) and there has been no natural recruitment recorded at the wild or translocations sites since monitoring began. The population at the wild site (site SU) has been supplemented by the Victorian Government Department of Energy, Environment and Climate Action (DEECA) in 2018 (approximately 170 propagated plants pers. comm. DEECA) and 2023 (approximately 160 propagated plants pers. comm. Karen Lester). In 2023, a new population of 144 plants was established using propagated plants at the site FE (Karen Lester pers. comm.). Previous research has determined the optimal size of plants and season of planting for translocations (Smith et al., 2009). All plants from this study are derived from the *ex-situ* living plant collection at the Royal Botanic Gardens Victoria.

*Diuris fragrantissima* plants have two channelled leaves to 20 cm long, which are produced during late summer and early autumn and maintained until late spring, with the plant persisting as a tuber through a short dormancy in summer (N. Reiter pers. obs.). A single scape is produced during the October-November flowering season (Jones, 2021). Each inflorescence has up to 12 nectar-less flowers (**Supplementary 1**) that are white with purple markings (VicFlora, 2024). Flowers are strongly fragrant with a similar sweet chocolate floral smell to *Arthropodium strictum* (Asparagaceae) on warm days (N. Reiter pers. obs.). Although pollinia are friable, the pollinia can be removed in their entirety via a basal viscidium and are clearly visible on pollinators to the naked eye. There were no other orchid species at these sites flowering concurrently that had pollinia with the same morphology as *D. fragrantissima*.

### Study sites

**Figure 1:**
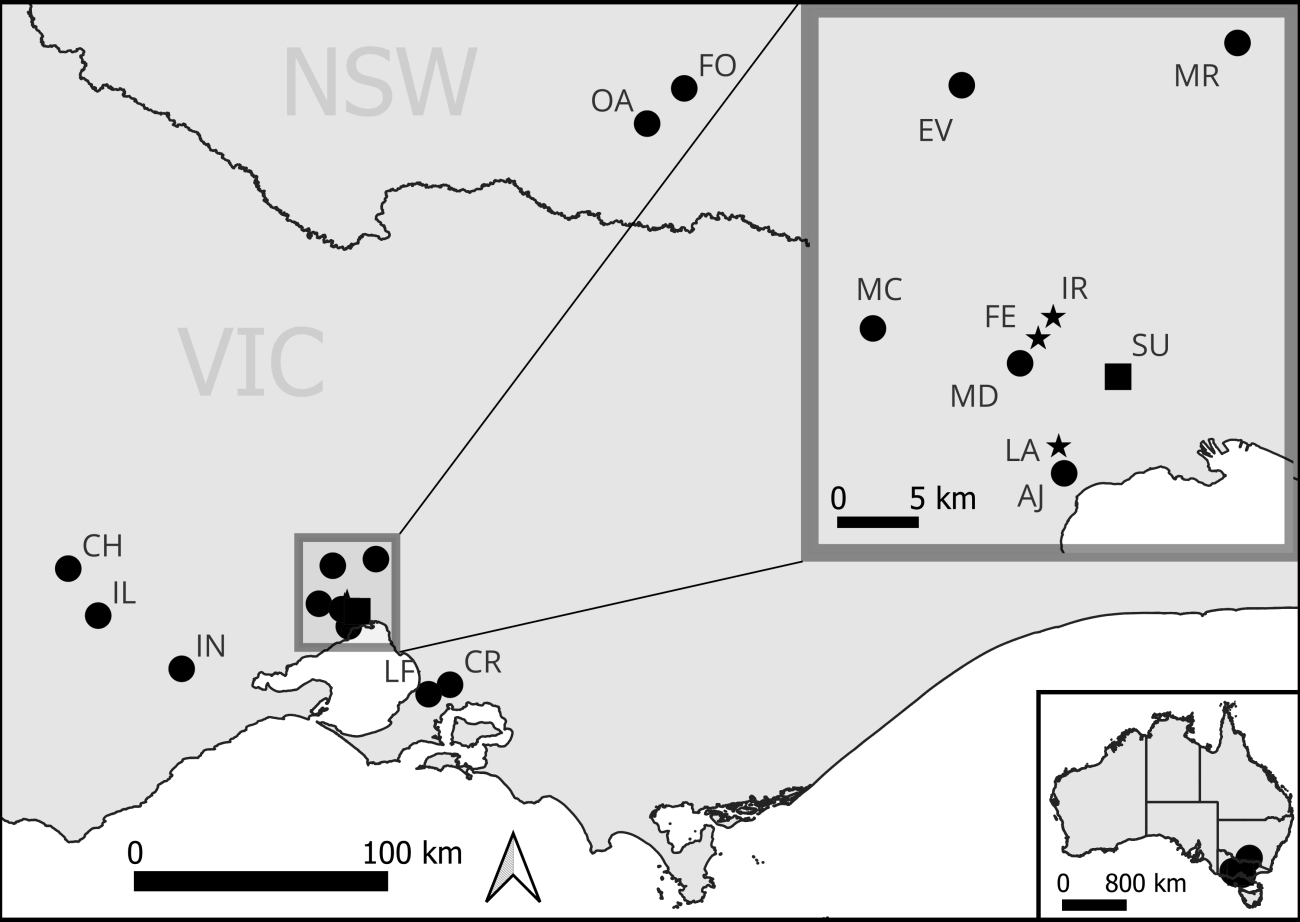
Map of study sites. Black square = the only remaining natural site (which has been supplemented) of *D. fragrantissima*. Black star = translocation sites of *D. fragrantissima*. Black circles= potential translocation sites of *D. fragrantissima*.

**Figure 2:**
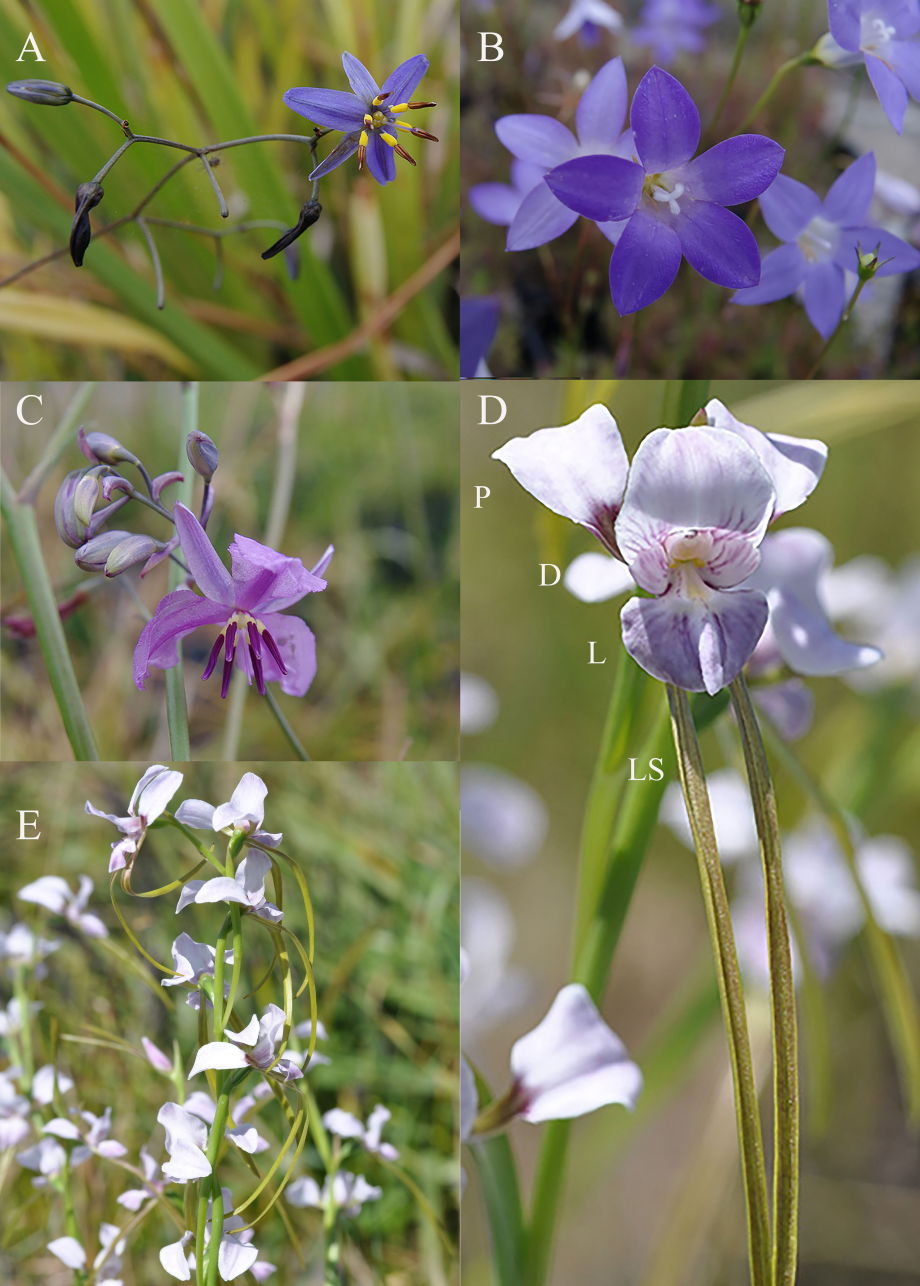
Species used to test if the addition of co-flowering species increases pollination in *Diuris fragrantissima*., **(A)** *Dianella reflexa* **(B)** *Wahlenbergia stricta*, **(C)** *Arthropodium strictum*, **(D)** the flower of *D. fragrantissima* (P = petal, D = dorsal sepal, L = labellum, LS = Lateral sepals) **(E)** inflorescence of *D*. *fragrantissima*.

In areas of remnant grassland (see **Table 1**, **Figure 1**), we undertook pollinator observations using cultivated *D. fragrantissima* and surveyed for the presence of the pollinators with either cultivated bait plants and/or vane traps depending on the location (**Table 1**). These sites included the one remaining wild site within the suburbs of Melbourne (Site SU, **Figure 1**), two existing translocation sites (LA and FE), and 13 potential translocation sites; IR, CH, IL, CR, MD, MR, MC, EV, OA, FO, LF, AJ and IN (**Figure 2**). Full site names have been withheld to protect the location of current and future populations. The sites were selected based on the presence of remnant grasslands or derived grasslands and secure land tenure. The majority of these locations are within the natural geographic range of *D. fragrantissima*. However, CH and IL are further west on the Victorian Volcanic Plain, CR and LF are on the east side of Melbourne, and OA and FO are in the Riverina region of southern New South Wales. The motivation for surveying for potential translocation sites outside of the geographic range of *D. fragrantissima*, but on suitable soils with similar vegetation, is that the majority of habitat within its known range has been destroyed for housing or agriculture, thereby limiting candidate translocation sites.

**Table 1:** Study sites for a pollination study of *D. fragrantissima*, denoting sites with pollinator observations, pollinator surveys with vane traps, and the collection of fruit set data.

Site abbreviation	Region	Pollinator obs.	Vane traps	Fruit set data
AJ	VVP		x	
CH	VVP	x	x	
CR	East Melbourne	x		x
EV	VVP	x		
FE*	VVP	x	x	x
FO	NSW	x		
IL	VVP	x	x	
IN	VVP	x		
IR	VVP	x	x	
LF	East Melbourne	x		
LA*	VVP	x	x	x
MC	VVP		x	
MD	VVP	x	x	
MR	VVP		x	
OA	NSW	x		
SU#	VVP	x	x	x

VVP refers to the Victorian Volcanic Plains. NSW refers to the state of New South Wales. * sites where plants have been introduced or supplemented via conservation translocation. # sites where there are wild plants.

### Pollinator identification and behaviour

Pollinator observations were undertaken using propagated flowers as bait for pollinators. Baiting for pollinators was originally used in studies of sexually deceptive orchids (Stoutamire, 1983; Peakall, 1990), where moving a picked flower to a new position in the landscape renewed the response of sexually attracted male pollinators. However, a similar approach can also be effective for orchids pollinated by food foraging pollinators, if many flowering scapes are used to increase the visual and/or chemical stimulus (e.g. Reiter et al., 2018; Scaccabarozzi et al., 2018; Phillips et al., 2020). Here, due to the Critically Endangered status of *D. fragrantissima*, we used flowering stems from *ex-situ* nursery plants as our bait plants (voucher specimens have been submitted to the National Herbarium of Victoria; MEL 2554603, MEL 2554604). Between 2019 and 2023, pollinator baiting was undertaken in October and November on sunny days >16°C, with low winds, between 10 am and 4 pm. Pollinator baiting was conducted with 6-8 stems held in a vial of water, giving 30-35 flowers in total. At each position in the landscape baiting was conducted for 6 minutes, before the bait flowers were shifted to a new position approximately 15 to 20 m away. Pollinator observations of *D. fragrantissima* were undertaken at the wild site (SU), the translocation site (LA), potential future translocation sites (IR, LF, CH, MD, MC, EV, IN, IL), and outside the natural geographic range but in intact grasslands (OA, FO) (**Table 1**). Pollinator observations were conducted for a total of 21 days (126 hrs) across the five years of the study.

To supplement direct pollinator observations, motion-activated game cameras for small insects were built using Raspberry Pi’s according to the github instructions of Whithead and Lanfear (https://github.com/roblanf/raspberrytrap). These methods were altered by substituting the recommended camera to a Raspberry Pi high-quality camera mount and associated 16 mm telephoto C-mount lens along with installing the realtime clock PCF8523 RTC module. Raspberry Pi’s were positioned on stakes in the field within 30 cm of the focal flowers. Flowers of *D. fragrantissima* were strapped to bamboo skewers to minimise movement in the wind. Video recordings using Raspberry Pi’s were undertaken in the same environmental conditions as the pollinator observations. At total of 156 hrs of observations were recorded. Two Raspberry Pi’s were used over a total of 12 hrs over 5 days at the FO and OA site in 2019 (**Table 1**). In 2020, seven Raspberry Pi’s were used for 6 hrs at site SU (total of 42 hours observation) and nine Raspberry Pi’s were used over 6 hrs at site LF (total of 54 hours observation) (**Table 1**). Over two days in 2021, eight Raspberry Pi’s were used for 6 hrs each at the site SU (total 48 hrs observation) (**Table 1**).

When recording pollinator behaviour, we noted pollen or pollinia removal and deposition. Here, we refer to pollen if only fragments of pollen were removed and pollinia if entire pollinia were removed. Deposition was noted by subsequent inspection of the flower’s stigma, while pollen or pollinia removal was via visual observations that showed pollinia were clearly removed by the animal when visiting the flower or pollen was partially removed during foraging behaviour. A subset of floral visitors was collected for identification. Pollinators were identified using published keys (Houston, 1981; Walker, 1986, 1995; Michener, 2007; Maynard, 2013; Leijs et al., 2017; Walker and Sparks, 2024).

### Surveying for pollinators at candidate translocation sites

To help determine which candidate pollinator species were present at the wild site (SU), existing translocation sites (LA and FE), and potential translocation sites (MC, MD, IL, MR, AX, FO and CH), in both 2022 and 2023 we collected bees using blue vane traps for five consecutive sunny days with low winds and temperatures >16°C during the flowering time of *D. fragrantissima* (October-November). Blue vane traps were used as they offer a consistent, repeatable level of survey effort, and because they have been shown to be effective for surveying the relevant bee genera in south-eastern Australia (Hall, 2018). We used the Blue vane traps available from Banfield Bio®, which are based on the description by Stephen and Rao (2005). The traps consist of a clear plastic collecting jar, 15 cm dia × 15 cm high, fitted with a blue fabricated polypropylene screw cap funnel into which two blue 24 x 13 cm (3 mm thick) polypropylene cross vanes are inserted. In 2022, two vane traps per site (100 m apart) were installed at the sites LA, MC, MD, IR, MR and AJ (**Figure 1**). In 2023, this trapping procedure was repeated at the sites LA and IR, and implemented at IL, FE, SU and CH. As our observations revealed that *D. fragrantissima* is bee-pollinated, only bees were identified from the vane traps.

### Does remnant grassland size affect bee diversity and abundance?

To test if bee diversity and abundance was affected by the spatial extent of the remnant area of grassland, we estimated the area of each remnant grassland where the bee community was surveyed. The perimeter of the remnant grassland was visually assessed, before Google Earth was used to determine the area of the grassland remnant. Grassland reserves were distinct from the surrounding environments, which were either houses, industrial estates or agricultural pasture. Methods for surveying for bees vary in their effectiveness for a given genus of bee (Prendergast et al., 2020), and not all methods (Raspberry pi’s, baiting with flowers and Blue vane traps) were deployed at all our sites. Therefore, we restricted the analysis on the effect of grassland remnant size on bee diversity and abundance to the Blue vane trap data from the ten sites where they were used (**Table 1**). We acknowledge that using a single method may not truly reflect the complete diversity of bees present at these sites.

### Does *D. fragrantissima* self-pollinate?

In 2021, 35 individuals of *D. fragrantissima* were placed in an insect-proof shade house to test for pollination without an insect vector, while 35 individuals were hand pollinated (6-12 flowers per plant) as a control to confirm fruit production in shade house conditions. Plants were observed for capsule formation.

### Fruit set at wild and translocation sites

The fruit set of wild *D. fragrantissima* over eight years (2006-2013) was recorded as very low (5.2% Duncan and Moloney, 2018). Subsequent pollination rates were recorded from 2018–2020 as 4.85 ± 3.03 (S.E) % (calculated from data in Duncan, 2021). Since then, there has been extensive habitat modification by DEECA and community members from the Australasian Native Orchid Society Victoria (at the wild site SU and LA), including removal of weed species and planting of a mixture of grassland forbs and grasses. Therefore, we quantified the pollination rates at site SU, LA and the recent translocation at FE. In addition, we quantified fruit set for an experimental population of potted plants at CR (see below) where pollinators were confirmed to be present. In 2023, the number of flowers and the number of flowers pollinated on each individual *D. fragrantissima* at each of these four sites were recorded. Percentage fruit set for a given year was calculated by dividing total number of fruits formed in a population by the total number of flowers.

### Is fruit set increased by hand pollination?

As reproduction of *D. fragrantissima* is overseen by land managers, we were not able to test the full extent of pollen limitation to fruit set. Ideally, this requires comparing fruit set between unmanipulated wild plants and wild plants where all flowers are pollinated by hand. However, it has been observed by land managers that in *D. fragrantissima*, hand-pollinations lead to higher rates of fruit set than typically encountered in wild plants. Therefore, we have made a comparison between wild rates of fruit set, and those resulting from hand-pollinations by land managers, which tests if there is some level of pollen limitation. In 2023, 38 *D. fragrantissima* flowers were hand-pollinated by land managers and DEECA, representing 13 flowers from 10 plants at Site FE (resulting in one plant with three pollinated flowers and another with two), 13 flowers from 10 plants at Site LA (three plants with two pollinated each), and 12 flowers from 11 plants at Site S (one plant with two pollinated). Each plant was identified by a tag, the pollen donor and recipient recorded, and the position on each plant of the flower pollinated was noted to discern between hand pollinations and natural pollinations. We compared the unmanipulated plants and artificially pollinated plants at each site to determine if hand pollinations led to an increase in the average number of flowers setting fruit set per plant.

### Is pollination enhanced through the presence of common co-flowering plants?

An experiment was undertaken at site CR to test if co-planting with commonly co-occurring native species is a potential management strategy for increasing reproductive success of *D. fragrantissima*. Plant species were selected for the experiment on the basis of (i) being bee pollinated (ii) naturally occurring at the wild site of *D. fragrantissima* (iii) flowering at the same time of year as *D. fragrantissima* (iv) being readily propagated at scale for any future plantings. This led to the selection of *Arthropodium strictum* (Asparagaceae), *Dianella reflexa* (Asphodelaceae) and *Wahlenbergia stricta* (Campanulaceae) (**Figure 2**), all of which provide a pollen reward and are therefore likely to primarily attract female bees. In the context of *D. fragrantissima*, this was considered appropriate as preliminary observations revealed that the majority of native visitors to *D. fragrantissima* were female bees (**Supplementary Table 1**). The site where the experiment took place was a derived grassland, which was originally the ecological vegetation community classified as Grassy Woodland (The State of Victoria Department of Sustainability and Environment 2004) prior to the removal of the eucalypt overstory. This site was selected as it was secure for the purposes of an experiment involving a threatened species in pots, and pollinator species of *D. fragrantissima* were present.

For this experiment, we had four treatments: 1) *D. fragrantissima* on their own, 2) *D. fragrantissima* with *W. stricta*, 3) *D. fragrantissima* with *A. strictum* and 4) *D. fragrantissima* with *D. reflexa*. Each *D. fragrantissima* replicate used in this experiment contained a single stem in a pot with 4-13 flowers. The co-flowering plants were also single plants in a pot, containing at least 10 flowers on each replicate plant. Co-flowering plants were placed 30 cm from the flowering *D. fragrantissima* plants. The treatments were set up in an experimental grid (108 x 8 m) in a strip of comparatively intact natural vegetation, which contained numerous forbs in addition to native and introduced grasses. The grid contained 27 rows, each containing one representative of three of the four treatments, with each replicate separated by 4 m. There were no other flowering plants within the grid experiment or within 10 m either side. To prevent consumption of experimental plants by the abundant herbivores at CR, each plant was surrounded with a plastic cage consisting of 5 cm mesh but with an open top. Based on previous studies of orchids pollinated by food foraging insects, these cages readily permit visitation by insects (e.g. Reiter et al., 2018, 2019a; Grinter, 2023).

To test if the experimental grid of potted plants led to increased pollination of *D. fragrantissima*, two additional control plots were established. These contained 12 *D. fragrantissima* each and were set up 23 m away from each end of the grid. Each block was 12 m by 8 m and plants were separated as they were in the above experiment by 4 m from each other. No co-flowering plants of the above species were present in the control plots.

Plants were checked daily and watered as required. Plants were left out for three weeks during the flowering period (which included 10 days of no rain and low winds) before being brought into a closed pollinator proof shade house where pollen deposition was assessed by visual inspection and confirmed by capsule formation.

### Statistical analysis

All analysis was conducted in R version 4.3.3 (R Core Team, 2023). A Generalised Linear Mixed Model (GLMM) in the glmmTMB package (Brooks et al., 2017) was used to test if the grassland remnant size affected the number of individual bees or the number of species of bee caught using vane traps. As surveys were conducted over two years, Year was treated as a random effect. We used a negative binomial distribution. For ‘number of bee species’, the control argument in glmmTMB() was used to adjust the maxfun parameter to specify the maximum number of function evaluations during optimization (1,000). The fit of the nbinom1 and nbinom2 distribution models was compared using AIC in the MuMIn package (Barton, 2012), with nbinom2 the better fit.

A Generalised Linear Model with a binomial error distribution was used to investigate the effect of co-flowering plants on the pollination of *D. fragrantissima*. As individual *D. fragrantissima* plants had multiple flowers, each plant was treated as a replicate. Each replicate included the number of flowers pollinated out of the total number of flowers available per individual.

A GLMM with a binomial error distribution (and quadrate as a random effect) was used to test the difference between control *D. fragrantissima* in the co-flowering grid (i.e. those without a co-plant) and those in the two nearby control quadrats.

For all analyses, model diagnostics were tested using the ‘DHARMa’ package (Hartig, 2022). Model comparisons were run using the package MuMIn (Barton, 2012). Model diagnostics showed no evidence of overdispersion or outliers, and good overall model fit based on residual plots.

## Results

### Pollinator identification and behaviour

**Figure 3:**
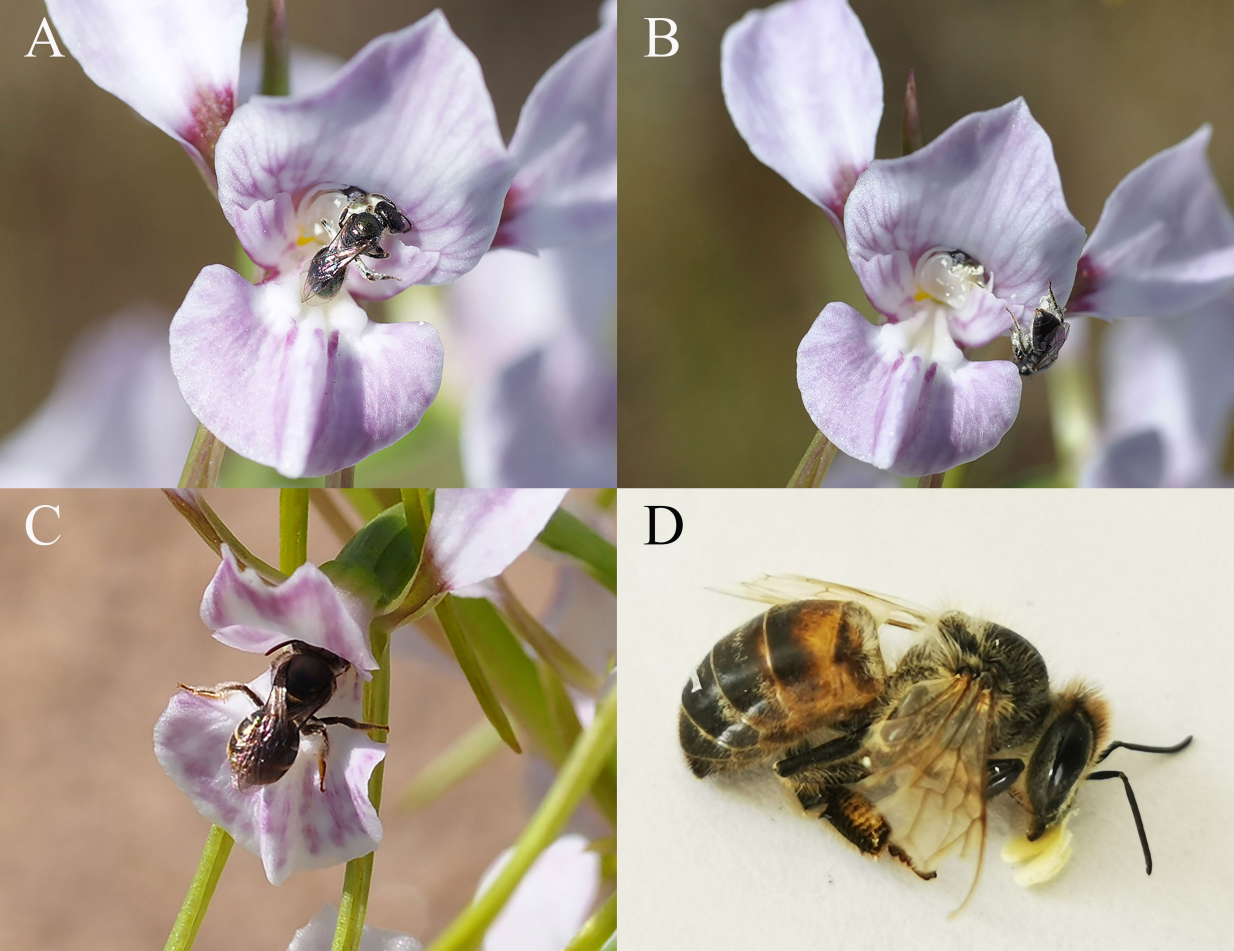
Floral visitors and pollinators of *Diuris fragrantissima* **(A)**: *Lasioglossum (Chilalictus) willsi* collecting pollen and **(B)** dislodging pollen onto the stigma*;* **(C)** *Lipotriches* (*Austronomia*) sp. entering *D*. *fragrantissima* flower contacting column and **(D)** *Apis mellifera* with pollinia on head.

From the combined direct observations and recordings with Raspberry Pi camera traps, five genera and 14 species of bee were observed landing on *D. fragrantissima* (**Table 2**; **Supplementary Table 1**). This included nine species of *Lasioglossum* (Halictidae), one species of *Amegilla* (Apidae), one species of *Lipotriches* (Halictidae), two species of *Lasioglossum (Homalictus)* (Halictidae) and the introduced *Apis mellifera* (Apidae). In total, 434 bees were observed landing on the labellum, 115 landed on the lateral sepals and 50 on the petals (**Table 2**, **Figure 3D** for flower structure, **Supplementary Table 1**). A total of 45 bees exhibited behaviour indicative of attempting to forage from the flower. Here, we refer to pollen when small amounts of friable pollen have been removed (through foraging) and pollinia when intact pollinia have been removed. In the larger bee species *Lipotriches (Austronomia)* sp., *Lasioglossum (Chilalictus) orbatum* and *Apis mellifera*, bees moved head-first to the base of the column and did not make any attempt to collect pollen (**Figures 3C, D**). This movement to the base of the column was associated with pollinia removal and deposition. Alternatively, the smaller bee species *Lasioglossum (Chilalictus) willsi*, *Lasioglossum* sp. and *Lasioglossum (Homalictus) sphecodoides*, used both their front and hind legs to attempt to remove the orchid pollen from behind the stigma, but did not appear to search for nectar or attempt to collect pollen from other parts of the flower (**Figures 3A, B**).

**Table 2:** Floral visitors and pollinators of *Diuris fragrantissima*.

Species	Family	A	L	F	C	PR	PD	Sites visiting
*Amegilla* sp.	Apidae	5	1	0	0	0	0	CH (2023), I (2023), IN (2023)
***Apis mellifera***	Apidae	1	205	5	0	14	8	I (2023), IN (2023), OA (2019), Fo (2019), Lan (2020)
*Hylaeus* sp.	Colletidae	0	1	1	0	0	0	MC (2022)
*Lasioglossum (Homalictus) holochlorus*	Halictidae	0	1	1	0	0	0	MC (2022)
*Lasioglossum (Homalictus)* sp.	Halictidae	1	59	5	0	0	0	S (2020, 2021), Lan (2021), MC (2022)
***Lasioglossum (Homalictus)*** ***sphecodoides***	Halictidae	0	6	2	0	1	1	S (2021), MC (2022)
*Lasioglossum* (*Chilalictus*) *clelandi*	Halictidae	0	2	0	1	0	0	MC (2022), CR (2023)
*Lasioglossum* (*Chilalictus*) *cognatum*	Halictidae	0	2	0	0	0	0	MC (2022)
*Lasioglossum* (*Chilalictus*) *erythrurum*	Halictidae	0	1	0	0	0	0	I (2023)
*Lasioglossum* (*Chilalictus*) *hemichalceum*	Halictidae	0	3	3	0	0	0	MC (2022)
*Lasioglossum* (*Chilalictus*) *lanarium*	Halictidae	0	1	1	0	0	0	I (2023)
*Lasioglossum* (*Chilalictus*) *mundulum*	Halictidae	0	13	0	0	0	0	CH (2023)
***Lasioglossum* (*Chilalictus*) *orbatum***	Halictidae	0	12	2	1	1	1	CH (2023), CR (2023)
*Lasioglossum* (*Chilalictus*) *sculpturatum*	Halictidae	0	2	0	0	0	0	IL (2023)
***Lasioglossum* (*Chilalictus*) *willsi***	Halictidae	0	8	0	0	1	0	FO (2019), SU (2020, 2021)
***Lasioglossum* (*Chilalictus*) sp.**	Halictidae	11	218	19	0	9	0	IL (2022, 2023), CH (2023), LA (2022), MC (2022), SU (2020)
***Lipotriches* (*Austronomia*) sp.**	Halictidae	0	64	6	3	6	1	CR (2023)

A, approached flower only; L, landed on the flower; F, exhibited foraging behaviour; C, contacted the column without removing or depositing pollen; PR, pollen or pollinia removed; PD, pollen or pollinia deposited. Species in bold were observed to remove or deposit pollen. Note, *Lasioglossum* sp. refers to individuals that were not identified to species level, as they were observed (or recorded on video) but not captured.

Thirty-two individual bees were observed removing pollinia and eleven were observed depositing pollinia (**Table 1**). Bees removing pollen or pollinia in their entirety were *Lipotriches (Austronomia)* sp. (N = 6), *Lasioglossum (Chilalictus) orbatum* (N = 1), *Lasioglossum (Chilalictus) willsi* (N = 1), *Lasioglossum* sp. (N = 9), *Lasioglossum (Homalictus)* sp*hecodoides* (N=1) and *A. mellifera* (N = 14). In *Lipotriches (Austronomia)* sp., *Lasioglossum (Chilalictus) orbatum and A. mellifera* (N = 14), pollinia was removed entirely and attached to the frontal region of the head (**Figures 3C, D**). Alternatively, the bees *Lasioglossum (Chilalictus) willsi*, *Lasioglossum* sp. and *Lasioglossum (Homalictus) sphecodoides* attempted to collect pollinia from the orchid. While dislodging the pollen with their legs, on occasion some friable pollen was lodged on the stigma, self-pollinating the orchid (**Figures 3A, B**). Based on these observations, *H.* sp*hecodoides* and *Lasioglossum (Chilalictus) willsi* were not considered effective pollinators. Those species that transferred pollinia between flowers or plants were *Lipotriches (Austronomia)* sp. (N = 1), *Lasioglossum (Chilalictus) orbatum* (N = 1) and *Apis mellifera* (N = 8). The numbers of observations of pollen deposition is expected to be low, as there were only flowering *D. fragrantissima* at sites SU and LA. For the bee species that transferred pollen between plants, the percentage of individuals visiting flowers that removed or deposited pollen was: *Lipotriches (Austronomia)* sp. (9.4% removal, 1.6% pollination, N = 64 visits); *Lasioglossum (Chilalictus) orbatum* (8.0% removal, 8.0% pollination, N = 12 visits); *Apis mellifera* (6.8% removal, 3.9% pollination, N = 205 visits).

### Surveying pollinators at suitable translocation sites

Across the ten sites, 288 bees were trapped from seven genera and 24 species (**Table 3**). The majority of species trapped (16) were from the genus *Lasioglossum*, with the most common species caught being *Lasioglossum (Chilalictus) lanarium* (N = 91). There was a difference in the proportion of males and female bees caught, with 83% of all bees captured being female (**Table 3**).

**Table 3:** A vane trap survey of the availability of confirmed pollinators of *Diuris fragrantissima* at the natural site * (SU), existing translocation sites # (LA and FE), and candidate translocation sites + (all other sites, **Figure 1**).

Site	Year	Bee species
SU*	2023	***Apis mellifera* (2)**
		*Lasioglossum (Chilalictus*) *imitans* F (6)
		*Lasioglossum (Chilalictus) lanarium* F(1), M(6)
		*Lasioglossum (Homalictus) sphecodoides* F (6)
		***Lipotriches (Austronomia)* sp. F(5)**
LA#	2022	***Apis mellifera* (3)**
		*Lasioglossum (Chilalictus) lanarium* M (1), F(1)
		*Lasioglossum (Homalictus)* sp*hecodoides* F (1)
	2023	*Amegilla (Notomegilla) murrayensis* F(1)
		*Lasioglossum (Chilalictus) clelandi* F(3)
		*Lasioglossum (Chilalictus) lanarium* F(1), M(2)
		***Lasioglossum (Chilalictus) orbatum* F (1)**
		*Lasioglossum (Homalictus) sphecodoides* F (1)
		***Lipotriches (Austronomia)* sp. F(1)**
FE#	2023	***Apis mellifera (*1)**
		*Lasioglossum (Chilalictus) clelandi* F(2)
		*Lasioglossum (Chilalictus) lanarium* F(1), M(1)
		*Lasioglossum (Chilalictus) mundulum* F(2)
		*Lasioglossum (Homalictus) sphecodoides* F(1)
IR+	2022	*Lasioglossum (Chilalictus) clelandi* F(1)
		*Lasioglossum (Chilalictus) lanarium* M (3), F(1)
		*Lasioglossum (Ctenonomia)* sp. F(1)
	2023	*Lasioglossum (Chilalictus) brazieri* F(8)
		*Lasioglossum (Chilalictus) lanarium* F(7), M(4)
		*Lasioglossum (Chilalictus) mundulum* F(1)
		*Lasioglossum (Ctenonomia)* sp. F(3)
		*Lasioglossum (Homalictus)* sp*hecodoides* F(2)
		*Lasioglossum (Parasphecodes) imitator* F(2)
		***Lipotriches (Austronomia)* sp F(1)**
MC+	2022	***Apis mellifera* (5)**
		*Lasioglossum (Homalictus) holochlorus* F(1)
		*Lasioglossum (Homalictus) sphecodoides* F(2)
		*Lasioglossum (Chilalictus) brazieri* F(17)
		*Lasioglossum (Chilalictus) clelandi* F(3)
		*Lasioglossum (Chilalictus) lanarium* F(28), M(5)
		*Lasioglossum (Chilalictus) repraesentans* F(1)
		*Lasioglossum (Chilalictus) sculpturatum* F(1)
		*Lasioglossum (Ctenonomia)* sp. F(1)
		*Lasioglossum* (*Parasphecodes*) *imitator* F(9)
MD+	2022	***Apis mellifera* (1)**
		*Lasioglossum (Homalictus) holochlorum* F(1)
		*Lasioglossum (Chilalictus) lanarium* M(1)
MR+	2022	***Apis mellifera* (3)**
		*Lasioglossum (Chilalictus) clelandi* F(4)
		*Lasioglossum (Chilalictus) lanarium* F(1), M (5)
		***Lipotriches (Austronomia)* sp. F(1)**
AJ+	2022	***Apis mellifera* (2)**
		*Euhesma* sp. F(8)
		*Lasioglossum (Chilalictus) lanarium* F(1)
		*Lasioglossum (Ctenonomia)* sp. F(1)
		*Lipotriches (Austronomia) australica* F(3)
		***Lipotriches (Austronomia)* sp. F(1)**
CH+	2023	***Apis mellifera (*1)**
		*Lasioglossum (Chilalictus) clelandi* F(42)
		*Lasioglossum (Chilalictus) cognatum* F(1)
		*Lasioglossum (Chilalictus) expansifrons* F(1)
		*Lasioglossum (Chilalictus) imitans* F(12)
		*Lasioglossum (Chilalictus) lanarium* F(12), M (2)
		*Lasioglossum (Chilalictus) mundulum* F(1)
		*Lasioglossum (Chilalictus) sculpturatum* F(1)
		*Leioproctus (Leioproctus) cupreus* F (1)
IL+	2023	*Amegilla (Notomegilla) murrayensis* F(3)
		*Lasioglossum (Chilalictus) clelandi* F(8)
		*Lasioglossum (Chilalictus) imitatans* F(1)
		*Lasioglossum (Chilalictus) lanarium* F (2), M (5)
		*Lasioglossum (Chilalictus) mundulum* F(1)
		*Lasioglossum (Parasphecodes) imitator* F(2)
		*Leioproctus (Exleycolletes)* sp A. F(1)
		*Lipotriches (Austronomia) australica* F(1)

Species in bold are those observed to remove or deposit pollen.

Of the ten sites surveyed with vane traps, five sites, including the natural site (SU), had the native pollinators of *D. fragrantissima* present (**Table 3**). *Lipotriches (Austronomia)* sp. was detected at five of the sites, one of which also had *Lasioglossum (Chilalictus) orbatum* (**Table 3**). It is worth noting that baiting for pollinators with *D. fragrantissima* flowers detected the pollinator *Lasioglossum (Chilalictus) orbatum* at the CH site while vane traps did not. In addition, baiting with flowers also detected the pollinators *Lasioglossum (Chilalictus) orbatum* and *Lipotriches (Austronomia)* sp. at the CR site, which did not have vane traps deployed. Using vane traps, *A. mellifera* was detected at eight out of 10 sites, with IR and IL being the only sites where this species was not detected.

### Does remnant grassland size affect bee diversity and abundance?

The ten sites surveyed with vane traps (**Table 3**) differed markedly in the species richness and number of individual bees caught with vane traps. At IL, neither the native nor introduced pollinators were detected using vane traps. At LA in 2022, IR in 2022 MD and MR only two or three bee species were caught. The highest number of species caught was at MC, CH and IL with eight or nine species. The lowest number of individual bees caught was LA (both years), IR (2023), MD (2022) and FE (2023), with less than 10 individual bees caught across a five-day period. The highest number of individual bees caught was at MC (73) and CH (74).

The area of remnant grassland had a significant effect on the total number of bees caught within a site (P < 0.001, N remnants = 10, **Table 4**). The area of remnant grassland also had a significant effect on the number of species of bee caught (P = 0.021, **Table 4**). Larger areas of remnant grassland were positively correlated with both total number of bees and number of bee species.

**Table 4:** Results of generalised linear mixed models investigating the influence of grassland area (km^2^) on total species of bees present or total number across 10 reserves.

Response variable	Explanatory variable	Est	S.E.	df	*X* ^2^	*P*
Species of Bee	Intercept	1.359	0.252			
	Grassland Area (km^2 *^)	0.147	0.064	8	4.913	**0.021**
Total number of bees	Intercept	2.288	0.383			
	Grassland Area (km^2*^)	0.384	0.058	8	13.306	**<0.001**

*Significance of the explanatory variables is based on likelihood-ratio tests (*X* ^2^) comparing models with and without the variable of interest. Significant variables (*P* < 0.05) are in bold and provided with estimates (Est.) and standard errors (S.E) for significant predictors.

### Fruit set at wild and translocation sites

The mean percentage of individual plants setting fruit across sites was 44.96 ± 4.30 (mean ± SE) % (N = 4 populations, 191 flowering plants in total) and the mean percentage of individual flowers setting fruit per population was 13.36 ± 2.22% (N = 4 populations, 985 flowers in total). The percentage of flowers setting fruit across the population varied from 19.9% at the wild site SU to 10.0% at the translocation site LA (**Supplementary Table 3**).

### Does *Diuris fragrantissima* self-pollinate?

All 35 individual plants (with 6-12 flowers each) that were hand pollinated in the shade house set seed. In the absence of hand pollination, no plants (35 plants with 6-12 flowers each) formed seed capsules.

### Does hand pollination increase fruit set?

At each site, hand pollination led to increased fruit set of wild plants relative to unmanipulated plants (Site S: supplemental pollination = 1.75 ± 0.16 fruits per plant (mean ± SE), plants unmanipulated = 0.06 ± 0.04 fruits per plant. Site F: supplemental pollination = 1.73 ± 0.24 fruits per plant, plants unmanipulated = 0.59 ± 0.11 fruits per plant. Site L: supplemental pollination = 1.63 ± 0.24 fruits per plant, plants unmanipulated = 0.65 ± 0.24 fruits per plant).

### Is pollination enhanced through the presence of common co-flowering plants?

Within the experimental grid the average pollination rate of *D. fragrantissima* without other flowering plants was 9.95 ± 3.05% (mean ± SE). When placed adjacent to *A. strictum* the pollination rate was 11.46 ± 4.05%, with *D. reflexa* it was 9.34 ± 2.78% and with *W. stricta* it was 18.73 ± 4.66%. Increased pollination success of *D. fragrantissima* was associated with the presence of co-flowering *W. stricta* (P<0.05, **Table 5**). There was no significant effect of any other co-flowering treatment. There was no significant difference between the pollination rate of *D. fragrantissima* without co-flowering plants within the experimental grid 9.95 ± 3.05% and those in the nearby plots 12.14 ± 4.18% (**Table 5**).

**Table 5:** Results of generalised linear models testing the influence of co-flowering plants on pollination success in *D. fragrantissima*.

Response Variable	Explanatory variable	Est	S. E.	df	z value	Pr(>|z|)
Pollination Success	Intercept	-2.204	0.263	104	8.367	<2e-16
	*Arthropodium strictum*	0.116	0.387		0.299	0.765
	*Dianella reflexa*	-0.128	0.423		-0.302	0.762
	*Wahlenbergia stricta*	0.715	0.350		2.040	**0.041**
	*D. fragrantissima* (alone)	0.304	0.376		0.809	0.419

Significant variables (P < 0.05) are in bold along with estimates (Est.) and standard errors (S.E.) for significant predictors.

## Discussion

Here, we have shown that the Critically Endangered orchid *Diuris fragrantissima* attracts a range of food-seeking bee species. While small members of the species-rich genus *Lasioglossum* were the native bees most frequently removing pollen, this typically resulted in geitonogamous pollen transfer. The only native species observed transferring pollen between flowers were the larger-bodied species *L. orbatum* and *Lipotriches* sp., though this was rarely recorded. A challenge with working with such a rare orchid species is that the chances of observing pollen deposition is low, as there are very few wild plants available to donate pollen. The potential habitat for *D. fragrantissima* is now just a series of remnant patches of grassland and the majority of grasslands surveyed in this study within the previous geographic range of *D. fragrantissima* were depauperate in bees. In addition to the bee species we observed, it is possible that other larger bee species may have originally contributed to reproduction of *D. fragrantissima* but have gone locally extinct.

Pollination of *D. fragrantissima* by *Lasioglossum* and *Lipotriches* contrasts with *D. brumalis* and *D. magnifica*, which are pollinated by *Trichocolletes* species that feed primarily on Fabaceae (Scaccabarozzi et al., 2018, 2020). Rather than pollination via mimicry of a guild of rewarding plants, as in *D. brumalis*, it is likely that *D. fragrantissima* is primarily using a deceptive strategy where the nectar-less flowers attract food-seeking bees through conspicuous floral signals but without close resemblance to other members of the flowering community (e.g. Ackerman, 1981; Nilsson, 1983; Fritz, 1990; Phillips and Batley, 2020). When the larger species of bee visited the flower, they moved to the base of the column, obscuring their head from view, meaning it was impossible to observe the mouthparts. However, in the absence of any attempts to collect pollen from the flower (e.g. *D. brumalis*, Scaccabarozzi et al., 2018), it is likely that they are searching for nectar when they visit *D. fragrantissima*. Alternatively, the smaller bees focused on attempting to collect the pollinia, but in attempting to do this, small amounts of pollen were deposited on the stigma leading to self-pollination (**Figures 3A, B**). Interestingly, while all species of bee primarily landed on the labellum, numerous individuals landed on the long, projecting lateral sepals, an unusual feature of subgenus *Diuris*, whose function remains unclear.

Despite a range of *Lasioglossum* species being detected at our study sites, in our baiting trials, the introduced *A. mellifera* was the species most often responsible for the removal and deposition of pollen. At the SU and LA populations of *D. fragrantissima*, we did not observe pollination by *A. mellifera* despite its presence at the sites. However, this may be due to bees experiencing the nectarless flowers of wild *D. fragrantissima* prior to our baiting for pollinators and therefore being less likely to respond. Though the proportion of *A. mellifera* removing pollen is much lower than observed for *Trichocolletes* bees in *Diuris magnifica* (25.5%, Scaccabarozzi et al., 2020), the rate of pollen removal and deposition by *A. mellfiera* is similar to the native bee species observed in *D. fragrantissima* and *Diuris brumalis* (Scaccabarozzi et al., 2018). While introduced *A. mellifera* are often less effective than natural pollinators in other plant species (e.g. Scaccabarozzi et al., 2018, 2024), because most orchids are pollen limited in a flowering season (Tremblay et al., 2005), *A. mellifera* can still contribute to plant reproduction. Globally, *Apis mellifera* visits a range of orchid species, typically those that are pollinated by bees rather than other pollination functional groups (see Ackerman et al., 2023). In Australia, records of *A. mellifera* visiting orchids are most frequent from species that are pollinated by food-seeking bees or wasps (e.g. Reiter et al., 2018, 2019a, 2019b; Scaccabarozzi et al., 2018, 2020), rather than species pollinated by sexual deception of wasps or fungus gnats (Phillips et al., 2017; Hayashi et al., 2025). At present, there are few reported cases of introduced bees making a substantial contribution to pollination of orchids. However, in the Andes of South America, introduced honeybees and bumblebees are highly effective pollinators of the orchid *Chloraea virescens*, replacing the declining native pollinators (Sanguinetti and Singer, 2014). Outside of orchids, there is evidence that *A. mellifera* can be effective pollinators of other Australian plants that were originally pollinated by bees (Gross, 2001) or a combination of birds and non-flying mammals (Gilpin et al., 2017; Wawrzyczek et al., 2024). Nonetheless, our result in *D. fragrantissima* is one of very few confirmed cases where introduced *A. mellifera* appear to be making an important contribution to the pollination of an endangered plant (see Caraballo-Ortiz et al., 2011; DeNittis and Meyer, 2022; Wawrzyczek et al., 2024).

The role of *A. mellifera* as a pollinator of *D. fragrantissima* in these small and often-degraded grassland remnants raises the possibility that for some Australian plant species *A. mellifera* may be an important pollinator in landscapes where native pollinators have declined. At the existing wild site in our study, bee diversity in the vane trap survey was generally low and was further reduced in smaller grassland remnants. For example, the most species rich sites in our study had a bee species richness that was similar to the mean recorded in agricultural habitats elsewhere in south-eastern Australia when using comparable survey methodology (e.g. Hall et al., 2019). However, the introduced *A. mellifera* is superabundant in southern Australia, reaching densities as high as 150 nests km^2^ in some wooded habitats (Paton, 1996; Oldroyd et al., 1997). Therefore, it is plausible that in areas where native pollinators have declined that *A. mellifera* may make a substantive contribution to pollination of some species. However, we argue that we should not be managing for *A. mellifera*, given that there is a high abundance of feral hives and that it likely competes for floral resources with other nectivorous insects (Wills et al., 1990). Further, the presence of introduced *A. mellifera* can lead to reduced fecundity of native bees (e.g. Paini and Roberts, 2005) and declines of hollow nesting birds via nest site competition (e.g. Johnstone and Kirkby, 2007).

Given that the abundant *A. mellifera* is contributing to pollination of *D. fragrantissima*, it is possible that pollinator availability may not impose a major limitation for selecting translocation sites for this species. However, in more intact areas of grassland, halictid bees may make a greater contribution to pollination of *D. fragrantissima* in addition to that of *A. mellifera*. As such, given that the diversity and overall abundance of native bees was greater in larger areas of remnant grassland, we predict that these would make for more effective translocation sites. Through their larger size, they may also be more buffered against declines of pollinator populations or invasion of weeds from adjoining areas.

In the year of our study, fruit set at populations of *D. fragrantissima* ranged between 10 and 20%. This is lower than the average of 20.7% for deceptive orchids in Tremblay et al. (2005) but exceeds those recorded for *D. brumalis* (approximately 2%; Scaccabarozzi et al., 2018) and *D. magnifica* (3.3%; Scaccabarozzi et al., 2020). Alternatively, the pollination rate for *D. fragrantissima* is far lower than observed in a closely related species in subgenus *Diuris*, *D. callitrophila* (30-60%, N. Reiter, unpublished data), which also grows in remnant grasslands, but in a different biogeographic region. In the co-planting experiment, pollination rates approximately doubled when *W. stricta* was positioned adjacent to *D. fragrantissima* plants (from 9.95% to 18.73%). While this result only applied to one of our three candidate species, other untested species of co-occurring plants may also be effective for increasing pollination of *D. fragrantissima*. For example, as a species that attracts pollinators via a pollen reward, *W. stricta* is unlikely to be effective for attracting male bees to *D. fragrantissima*, though it is unclear how much they contribute to pollination. It would be interesting to test if co-planting benefits are associated with plants that are visually conspicuous or provide larger rewards.

From a management perspective, the question remains if the beneficial effect of *W. stricta* would apply once the *D. fragrantissima* begin to reproduce at an introduction site, with some seedlings likely to be distant from an individual *W. stricta*. As pollinator attraction appears not to be based on floral mimicry, the increased pollination to *D. fragrantissima* could arise because *W. stricta* increases the conspicuousness of the overall floral display, leading to increased attraction of pollinators to the pair of plants. Alternatively, pollinators may focus their foraging on *W. stricta* and incidentally visit *D. fragrantissima*. In either of these scenarios the benefit of co-planting is likely to operate on very small scales (e.g. Gumbert and Kunze, 2001). Benefits to the orchid population in general may arise from a magnet effect, where pollinators are lured to a patch of vegetation by the aggregation of *W. stricta* (e.g. Johnson and Steiner, 2003), meaning that the orchid may benefit through increased pollinator visitation even if not in the immediate vicinity of the rewarding plants. In the present study, the other treatments in the experimental grid and the adjoining control plots did not differ in pollination rate, only those that were next to *W. stricta*, which suggests that a magnet effect was not operating. However, if *W. stricta* were supplemented at a site in larger numbers, a magnet effect may occur.

In conclusion, the Critically Endangered orchid *D. fragrantissima* appears to be pollinated by a small number of species of halictid bees and the introduced *A. mellifera*. The diversity of bees in these areas of remnant grassland is generally low, meaning that in remnants where native pollinators have declined *A. mellifera* may make a substantial contribution to the pollination of *D. fragrantissima*. We recommend prioritising larger remnants for conservation translocations of *D. fragrantissima*, as they support larger populations of *Lasioglossum* and *Lipotriches* bees, and are more likely to buffered against degradation in a fragmented landscape. As most native habitat remnants on the VVP are highly degraded (Morgan and Williams, 2015), selection of future translocation sites will need to factor in appropriate management for grassland habitat, including control of introduced herbivores (Olff and Ritchie, 1998), appropriate fire regimes (Lunt, 1994; 2012) and reduction of weed cover, particularly those that have allelopathic affects or reduce inter tussock spaces for recruitment (Humphries et al., 2021). In addition, site selection should incorporate which part of the VVP will be most climatically suitable for *D. fragrantissima* following predicted climate change. Once suitable sites are selected, we demonstrate the plausibility of co-planting with rewarding plants to increase reproductive rates in *D. fragrantissima*. It will be interesting to test if this applies to other native food plants, and if this is a useful strategy for other threatened herbs.

## Data Availability

The raw data supporting the conclusions of this article will be made available by the authors, without undue reservation.
