# Supplementary material for: Restoring a critically endangered grassland orchid by co-planting to improve pollination and selecting sites based on pollinator availability
Source: Front Plant Sci. 2025 May 23;16:1566543. doi: 10.3389/fpls.2025.1566543 (PMC12141344; doi:10.3389/fpls.2025.1566543)
Supplement: Supplementary file 1 [file Table1.docx]

Supplementary Methods 1

***Testing for the presence of labellum surface sugars using GC-MS analysis***

We used the sampling methodology of Reiter et al., (2018), which was developed to test for the presence of sugars in flowers that lack droplets of nectar. A total of 10 flowers of *Diuris fragrantissima* (1 flower per individual) were sampled from the RBGV living *ex situ* collection and placed into a glasshouse at 20 °C for 3 h prior to sampling. In short, for each of ten plants, three drops of an aqueous solution of ribitol (5 mL, internal standard, 0.20 mg/mL) were added with a glass syringe onto three separate parts of the labellum of each flower. The aqueous solution was subsequently collected with microcapillary tubes (5 mL) and immediately transferred to GC vials (2 mL) with inserts (50 μL). The three aliquots from each flower were combined in the same vial. Solutions taken from the flower were stored in a −20 °C freezer until analysis. For further description of the GC methodology see Reiter et al., (2018) and Lisec et al., (2006).

Supplementary Table 1: Floral visitors and pollinators of *Diuris fragrantissima*, broken down by study site and year of observation. Y: year of observation, S: study site, A: approached without landing, L: landed on labellum, S: landed on sepals, P: Pollen removal, F: exhibited feeding or foraging behaviour head down and/or pollinia gathering, C: column contact, PD: pollinia deposition, PR: Pollinia removal. * Not identified to species identified by observation, **Raspberry Pi video rather than human observation.

| **Species** | **Y** | **S** | **A** | **L** | **S** | **P** | **F** | **C** | **PD** | **PR** |
| --- | --- | --- | --- | --- | --- | --- | --- | --- | --- | --- |
| *Lasioglossum* *(Chilalictus)* sp. * | 2023 | IL |  | 30 | 15 | 1 | 13 |  |  | 1 |
| *Lasioglossum (Chilalictus)* *erythrurum* (F) | 2023 | IL |  | 1 |  |  |  |  |  |  |
| *Lasioglossum (Chilalictus)* *sculpturatum* (F) | 2023 | IL |  | 2 |  |  |  |  |  |  |
| *Lasioglossum* *(Chilalictus) lanarium* (M) | 2023 | IL |  | 1 |  |  | 1 |  |  |  |
| *Amegill*a sp* | 2023 | IL | 2 |  |  |  |  |  |  |  |
| *Apis* *mellifera* | 2023 | IL | 1 |  |  |  |  |  |  |  |
|  |  |  |  |  |  |  |  |  |  |  |
| *Lasioglossum* *(Chilalictus)* sp. * | 2022 | IR |  | 4 |  |  |  |  |  |  |
|  |  |  |  |  |  |  |  |  |  |  |
| *Lasioglossum* *(Chilalictus)* sp. * | 2023 | CH | 9 | 28 | 18 | 1 | 4 |  |  |  |
| *Lasioglossum* *(Chilalictus)* *clelandi* (F) | 2023 | CH |  | 1 |  |  |  |  |  |  |
| *Lasioglossum (Chilalictus)* *mundulum* (F) | 2023 | CH |  | 8 | 5 |  |  |  |  |  |
| ***Lasioglossum* (*Chilalictus*) *orbatum* (F)** | 2023 | **CH** |  | 4 |  |  | 2 |  |  |  |
| *Amegilla* sp.* | 2023 | CH | 2 |  |  |  |  |  |  |  |
|  |  |  |  |  |  |  |  |  |  |  |
| *Amegilla* sp.* | 2023 | IN | 1 | 1 |  |  |  |  |  |  |
| *Apis mellifera* | 2023 | IN |  | 6 |  |  | 5 |  |  |  |
|  |  |  |  |  |  |  |  |  |  |  |
| ***Lipotriches* (*Austronomia*) sp. (F)** | 2023 | **CR** |  | 46 | 18 | 0 | 6 | 3 | 1 | 6 |
| ***Lasioglossum (Chilalictus) orbatum* (M)** | 2023 | **CR** |  | 6 |  | 2 |  | 1 | 1 | 1 |
|  |  |  |  |  |  |  |  |  |  |  |
| *Lasioglossum* *(Chilalictus)* sp. * | 2022 | LA |  | 3 |  |  |  |  |  |  |
|  |  |  |  |  |  |  |  |  |  |  |
| *Lasioglossum* *(Chilalictus)* sp. * | 2022 | MC |  | 27 | 2 |  | 2 |  |  |  |
| *Lasioglossum (Chilalictus*) *cognatum*(F) | 2022 | MC |  | 2 |  |  |  |  |  |  |
| *Lasioglossum (Chilalictus) hemichalceum* (F) | 2022 | MC |  | 3 |  |  | 3 |  |  |  |
| *Lasioglossum* *(Chilalictus)* *clelandi* (F) | 2022 | MC |  | 1 |  |  |  | 1 |  |  |
| *Homalictus* sp. * | 2022 | MC |  | 16 | 2 |  | 1 |  |  |  |
| *Homalictus holochlorus* (F) | 2022 | MC |  | 1 |  |  | 1 |  |  |  |
| *Homalictus sphecodoides* (F) | 2022 | MC |  | 1 |  |  | 1 |  |  |  |
| *Hylaeus* sp. | 2022 | MC |  | 1 |  |  | 1 |  |  |  |
|  |  |  |  |  |  |  |  |  |  |  |
| *Lasioglossum (Chilalictus)* *willsi* (F) | 2021 | SU |  | 2 | 2 |  |  |  |  |  |
| ***Homalictus sphecodoides* (F)** | **2021** | **SU** |  | **3** | **2** |  | **1** |  | **1** | **1** |
| *Homalictus* sp. (F) | 2021 | SU |  | 22 | 8 |  | 4 |  |  |  |
| *Homalictus* sp. (F) | 2020 | SU |  |  |  |  |  |  |  |  |
| ***Lasioglossum* (*Chilalictus*) *willsi* (F)** | **2020** | **SU** |  | **1** |  |  |  |  | **1** | **1** |
| *Lasioglossum* *(Chilalictus)* sp. ** | 2020 | SU | 2 | 38 | 23 | 28 |  |  |  | 8 |
|  |  |  |  |  |  |  |  |  |  |  |
| *Homalictus* sp. (F) | 2021 | LF | 1 | 8 | 1 | 2 |  |  |  |  |
| *Apis mellifera* ** | 2020 | LF |  | 4 |  |  |  |  |  |  |
|  |  |  |  |  |  |  |  |  |  |  |
| *Apis mellifera* | 2019 | OA |  | 12 | 3 | 1 |  |  |  |  |
| *Apis mellifera* ** | 2019 | OA |  | 36 | 7 | 8 |  |  |  |  |
|  |  |  |  |  |  |  |  |  |  |  |
| ***Apis mellifera*** | **2019** | **FO** |  | **17** | **1** |  |  |  | **1** | **3** |
| *Lasioglossum (Chilalictus)* *willsi* | 2019 | FO |  | 1 | 2 |  |  |  |  |  |
| ***Apis mellifera* **** | **2019** | **FO** |  | **97** | **6** | **7** |  |  | **7** | **11** |

Supplementary Table 2: Bees caught in vane traps over five sunny days, during the flowering period of *D. fragrantissima* at potential translocation sites with similar vegetation in 2022 and 2023. *Wild *D. fragrantissima* site, **Site where *D. fragrantissima* previously translocated. Bold highlights are confirmed pollinator species. N sp. refers to the total number of bee species recorded at the site, N indiv. refers to the total number of individual bees recorded at the site.

| **Site** | **Year** | **Species** | **N sp.** | **N indiv.** | **Remnant area (ha)** |
| --- | --- | --- | --- | --- | --- |
| **LA | 2022 | ***Apis mellifera* (3)** | 3 | 6 | 0.5 |
|  |  | *Lasioglossum (Chilalictus) lanarium* M (1), F (1) |  |  |  |
|  |  | *Lasioglossum (Homalictus) sphecodoides* F (1) |  |  |  |
|  | 2023 | *Amegilla* *(Notomegilla) murrayensis* F (1) | 6 | 10 | 0.5 |
|  |  | *Lasioglossum (Chilalictus) clelandi* F (3) |  |  |  |
|  |  | *Lasioglossum (Chilalictus) lanarium* F (1), M (2) |  |  |  |
|  |  | ***Lasioglossum (Chilalictus) orbatum* F (1)** |  |  |  |
|  |  | *Lasioglossum (Homalictus) sphecodoides* F (1) |  |  |  |
|  |  | ***Lipotriches (Austronomia)* sp. F (1)** |  |  |  |
| IR | 2022 | *Lasioglossum (Chilalictus) clelandi* F (1) | 3 | 6 | 0.32 |
|  |  | *Lasioglossum (Chilalictus) lanarium* M (3), F (1) |  |  |  |
|  |  | *Lasioglossum (Ctenonomia)* sp*.* F (1) |  |  |  |
|  | 2023 | *Lasioglossum (Chilalictus) brazieri* F (8) | 7 | 28 | 0.32 |
|  |  | *Lasioglossum (Chilalictus) lanarium* F (7), M (4) |  |  |  |
|  |  | *Lasioglossum (Chilalictus) mundulum* F (1) |  |  |  |
|  |  | *Lasioglossum (Ctenonomia)* sp. F (3) |  |  |  |
|  |  | *Lasioglossum (Homalictus) sphecodoides* F (2) |  |  |  |
|  |  | *Lasioglossum (Parasphecodes) imitator* F (2) |  |  |  |
|  |  | ***Lipotriches (Austronomia)* sp. F (1)** |  |  |  |
| MC | 2022 | ***Apis mellifera* (5)** | 10 | 73 | 6.35 |
|  |  | *Homalictus holochlorus* F (1) |  |  |  |
|  |  | *Homalictus sphecodoides* F (2) |  |  |  |
|  |  | *Lasioglossum (Chilalictus) brazieri* F (17) |  |  |  |
|  |  | *Lasioglossum (Chilalictus) clelandi* F (3) |  |  |  |
|  |  | *Lasioglossum (Chilalictus) lanarium* F (28), M (5) |  |  |  |
|  |  | *Lasioglossum (Chilalictus) repraesentans* F (1) |  |  |  |
|  |  | *Lasioglossum (Chilalictus) sculpturatum* F (1) |  |  |  |
|  |  | *Lasioglossum (Ctenonomia)* sp*.* F (1) |  |  |  |
|  |  | *Lasioglossum (Parasphecodes) imitator* F (9) |  |  |  |
| MD | 2022 | ***Apis mellifera* (1)** | 3 | 3 | 0.28 |
|  |  | *Lasioglossum (Homalictus) holochlorum* F (1) |  |  |  |
|  |  | *Lasioglossum (Chilalictus) lanarium* M (1) |  |  |  |
| MR | 2022 | ***Apis mellifera* (3)** | 4 | 14 | 3.18 |
|  |  | *Lasioglossum (Chilalictus) clelandi* F (4) |  |  |  |
|  |  | *Lasioglossum (Chilalictus) lanarium* F (1), M (5) |  |  |  |
|  |  | ***Lipotriches (Austronomia)* sp*.* F (1)** |  |  |  |
| AJ | 2022 | ***Apis mellifera* (2)** | 6 | 16 | 0.72 |
|  |  | *Euhesma sp.* F (8) |  |  |  |
|  |  | *Lasioglossum (Chilalictus) lanarium* F (1) |  |  |  |
|  |  | *Lasioglossum (Ctenonomia) sp.* F (1) |  |  |  |
|  |  | *Lipotriches (Austronomia) australica* F (3) |  |  |  |
|  |  | ***Lipotriches (Austronomia)* sp*.* F (1)** |  |  |  |
| *SU | 2023 | ***Apis mellifera* (2)** | 5 | 25 | 0.02 |
|  |  | *Lasioglossum (Chilalictus) imitans* F (6) |  |  |  |
|  |  | *Lasioglossum (Chilalictus) lanarium* F (1), M (6) |  |  |  |
|  |  | *Lasioglossum (Homalictus) sphecodoides* F (6) |  |  |  |
|  |  | ***Lipotriches (Austronomia)* sp*.* F (5)** |  |  |  |
| CH | 2023 | ***Apis mellifera* (1)** | 9 | 74 | 3.8 |
|  |  | *Lasioglossum (Chilalictus) clelandi* F (42) |  |  |  |
|  |  | *Lasioglossum (Chilalictus) cognatum* F (1) |  |  |  |
|  |  | *Lasioglossum (Chilalictus) expansifrons* F (1) |  |  |  |
|  |  | *Lasioglossum (Chilalictus) imitans* F (12) |  |  |  |
|  |  | *Lasioglossum (Chilalictus) lanarium* F (12), M (2) |  |  |  |
|  |  | *Lasioglossum (Chilalictus) mundulum* F (1) |  |  |  |
|  |  | *Lasioglossum (Chilalictus) sculpturatum* F (1) |  |  |  |
|  |  | *Leioproctus (Leioproctus) cupreus* F (1) |  |  |  |
| **FE | 2023 | ***Apis mellifera* (1)** | 5 | 8 | 0.03 |
|  |  | *Lasioglossum (Chilalictus) clelandi* F (2) |  |  |  |
|  |  | *Lasioglossum (Chilalictus) lanarium* F (1), M (1) |  |  |  |
|  |  | *Lasioglossum (Chilalictus) mundulum* F (2) |  |  |  |
|  |  | *Lasioglossum (Homalictus) sphecodoides F* (1) |  |  |  |
| IL | 2023 | *Amegilla murrayensis* F (3) | 8 | 24 | 1.14 |
|  |  | *Lasioglossum (Chilalictus) clelandi* F (8) |  |  |  |
|  |  | *Lasioglossum (Chilalictus) imitatans* F (1) |  |  |  |
|  |  | *Lasioglossum (Chilalictus) lanarium* F (2), M (5) |  |  |  |
|  |  | *Lasioglossum (Chilalictus) mundulum* F (1) |  |  |  |
|  |  | *Lasioglossum (Parasphecodes) imitator* F (2) |  |  |  |
|  |  | *Leioproctus (Exleycolletes)* sp*.* F (1) |  |  |  |
|  |  | *Lipotriches (Austronomia) australica* F (1) |  |  |  |

Supplementary Table 3: Rates of fruit set in wild and translocated populations of *Diuris fragrantissima* in 2023. The Cranbourne site refers to the plots adjacent to the experimental grid of co-flowering plants.

| **Site** | ***N* individuals pollinated** | ***N* individuals Flowering** | **% plants setting fruit** | ***N* flowers pollinated** | ***N* flowers** | **% flowers setting fruit** |
| --- | --- | --- | --- | --- | --- | --- |
| SU | 44 | 77 | 57.1 | 69 | 346 | 19.9 |
| LA | 14 | 33 | 42.4 | 20 | 200 | 10.0 |
| FE | 27 | 62 | 43.5 | 36 | 316 | 11.4 |
| CR | 7 | 19 | 36.84 | 16 | 123 | 12.14 |
